# Medical treatment of pediatric urolithiasis

**DOI:** 10.1007/s00467-007-0740-7

**Published:** 2008-02-14

**Authors:** Uri S. Alon

**Affiliations:** Bone and Mineral Disorders Clinic, Pediatric Nephrology, Children’s Mercy Hospital and Clinics, University of Missouri at Kansas City, 2401 Gillham Road, Kansas City, MO 64108 USA

**Keywords:** Hypercalciuria, Hypocitraturia, Oxaluria, Potassium, Sodium, Urolithiasis

## Abstract

In recent years the incidence of pediatric stone disease has increased several fold, mostly due to hypercalciuria and hypocitraturia. The goal of medical treatment is to protect the patient from formation of new stones and expansion of existing ones. The non-pharmacological means to address stone disease include high fluid intake and, frequently, modification of nutritional habits. The pharmacological treatment is based on the chemical composition of the stone and the biochemical abnormalities causing its formation; hence, chemical analysis of the stone, urine and blood is of paramount importance and should be done when the first stone is detected. This review discusses the current options of medical treatment of pediatric urolithiasis.

## Introduction

Most recent studies report that the incidence of kidney and ureteral stones has increased significantly in both adults and children [1–3]. Others, however, report no change in incidence [4]. Most stones in children are composed of calcium oxalate and calcium phosphate, or a combination of the two. The two more common pathophysiologic mechanisms of these stones are idiopathic hypercalciuria and hypocitraturia [3, 5]. Assuming that the genetic pool of patients predisposed to these conditions has not changed dramatically, one can speculate that the main reason for the rise in incidence is mostly due to environmental factors. The latter possibly include changes in nutritional habits, regional climate changes (http://en.wikipedia.org/wiki/Global_warming) and decreased fluid intake.

Unfortunately, chemolysis is effective only in a small fraction of specific stones, like alkali therapy for uric acid stones and when combined with thiols (see below) for cystine stones, and urine acidification for struvite stones. Furthermore, in most cases, chemolysis is used for local irrigation, although, on occasions, it can be used as systemic therapy [6]. Therefore, in almost all cases, removal of existing stones remains in the domain of the urologist. The medical treatment of urolithiasis is aimed at protecting the patient from further growth of existing stones and development of new stones, thus decreasing morbidity and the need for surgical intervention. Hence, under these circumstances, medical treatment, in practicality, means prevention. To achieve this goal it is important for one to obtain stone analysis, if feasible, and urine chemistry analysis as early as possible (Table 1). In essence, this challenging treatment has two main avenues which operate in concert: the non-pharmacological one and the pharmacological one (Table 2). This review addresses these two aspects of the medical treatment of the more common stone disorders in childhood and adolescence.

**Table 1:** Normal values for 24 h urine

Chemical component	Value
Calcium	< 4 mg (0.1 mmol)/kg per 24 hours
Sodium	<3 mEq (3 mmol)/kg per 24 hours
Potassium	>3 mEq (3 mmol)/kg per 24 hours
Magnesium	>88 mg (44 mmol)/1.73 m^2^ per 24 hours
Citrate	>180 mg (94 μmol/g (8.84 mmol) creatinine
Oxalate	<52 mg (593 mmol)/1.73 m^2^ per 24 hours
	<2 mg (23 mmol)/kg per 24 hours
Cystine	<60 mg (0.5 mmol)/1.73 m^2^ per 24 hours
Uric Acid	<815 mg (4.9 mmol)/1.73 m^2^ per 24 hours
	<35 mg (0.21 mmol)/kg per 24 hours
Xanthine	30-90 μg (20–60 μmol)/24 h

**Table 2:** Medications commonly used in the treatment of urolithiasis

Drug name	Dosage (oral)	Formulations	Indications
Allopurinol	10 mg/kg per day. Single or divided 2–3 doses	Tablet: 100 mg, 300 mg. Oral solution: can be made from tablets	Hyperuricosuria
Captopril	0.5–1.5 mg/kg per dose. Given 2–4 times per day (lower starting dose in infants)	Tablet: 12.5 mg, 25 mg, 50 mg. Oral solution: 1 mg/ml may be prepared	Cystinuria
Chlorothiazide	10–20 mg/kg per day divided 1–2 doses	Tablet: 250 mg, 500 mg. Oral solution: 250 mg/5 ml	Hypercalciuria, hyperoxaluria
Hydrochlorothiazide (HCTZ)	1–2 mg/kg per day divided 1–2 doses	Tablet: 25 mg, 50 mg. Oral solution: 50 mg/5 ml	Hypercalciuria, hyperoxaluria
Moduretic	Based on HCTZ	Tablet: (HCTZ 50 mg/amiloride 5 mg)	Hypercalciuria, hyperoxaluria
Potassium (K) citrate	0.5 – 1.5 mEq K/kg per day. Divided 2 doses (tablets) or 3 doses (liquid) (titrate higher if needed)	Tablet: 5 mEq K, 10 mEq K. Solution: 2 mEq K/ml	Hypocitraturia, hypercalciuria, hyperuricosuria, cystinuria
Pyridoxine (vitamin B_6_)	25–200 mg/day, once daily (titrated to effect with urine oxalate levels)	Tablet: 25 mg, 50 mg	Primary hyperoxaluria
Tiopronin	15 mg/kg per day divided 3 doses	Tablet: 100 mg	Cystinuria
D-Penicillamine	30 mg/kg per day divided 4 doses, half at night	Capsule: 125 mg, 250 mg	Cystinuria

## Non-pharmacological intervention

### Fluids

The universal and probably most important component of the treatment of all kinds of kidney stones is increased urine volume, thereby decreasing solute concentration and, consequently, supersaturation [7]. As shown by Miller and Stapleton [8] children with urolithiasis tend to have a lower 24 hour urine volume than do controls. Lande et al. [9], studying 32 children with urolithiasis, showed that urine flow of >1 ml/kg per hour, almost eliminated the risk of supersaturation for calcium oxalate, calcium phosphate and uric acid, thus protecting from the formation of the corresponding kidney stones. In a child weighing 40 kg this translates to 960 ml urine/24 h. In patients with cystinuria the required urine flow may be even higher (see below), and, in other cases, such as primary xanthinuria, high fluid intake is the only therapy available. In most patients, for them to achieve the desired urine output, an increase in oral fluid intake is required. Although no studies were done in children, in a large scale epidemiologic study in women, Curhan et al. [7] found that coffee, tea and alcoholic beverages decreased the risk of stone formation, whereas grapefruit juice increased it. In a previous study in men, the same group showed an increased risk for stone formation associated with grapefruit and apple juice [10]. The reason for the association of these two beverages with increased stone risk is unknown. Milk, other fruit juices, and water do not have an effect in either direction, therefore they (plus tea) become the beverages of choice for children. Of note, when one is discussing milk with patients and their families, the recommended intake should be within the frame of the recommended dietary allowance (RDA) of calcium and protein (vide infra). In adults, some studies found all types of soda, with caffeine, without caffeine, conventional sweet soda and diet soda, to be adequate for use as beverages [7, 10]. However, others found lower rates of stone recurrence in those discontinuing soda drinks, especially when the drinks contained phosphoric acid [11]. If one wants to add an advantage to the drink used, then those beverages containing alkali are regarded to be superior to acidic fluids, as discussed below [6, 12, 13].

Unfortunately, it seems that, in children, the recommendation for high fluid intake has only limited success. As recently shown, even after they had been provided with the recommendation of high fluid intake, children with calcium stones continued to have high urine specific gravity, used as a surrogate for urine volume, that was not different from that of healthy, age-matched, controls [5]. This means that further efforts should be made in finding better educational tools and practices to instill the habit of high fluid intake. On the other hand, all the time such a goal has not been achieved, other preventive means should be utilized.

### Nutrition

The other main non-pharmacological intervention involves the child’s nutrition. Multiple studies have shown the coupling between urinary sodium and calcium excretion and, consequently, between high dietary sodium intake, which mostly finds its way to the urine, and hypercalciuria [14]. Moreover, in a recent study, Frassetto et al. [15] showed that dietary sodium chloride, by means of chloride, induces low-grade metabolic acidosis which, as discussed below, adversely affects bone status and promotes stone formation. For the majority of patients with calcium stones and most other types of stones, dietary sodium adjustment indicates a significant reduction in its intake. This is due to the fact that, in industrialized countries, and mostly due to food processing, the population consumes sodium in amounts far exceeding the physiologic needs. Hence, “sodium restriction”, in essence, should reflect changing the habits of high sodium intake to that of optimal sodium intake. The Food and Nutrition Board of the Institute of Medicine, National Academy of Sciences, states that the optimal daily intake of sodium at ages 4–8 years is 1.2 g and, at ages 9–18 years, is 1.5 g, and the upper limit values (namely maximal amounts not to pose health risks) are 1.9 g and 2.3 g, respectively (http://dietary-supplements.info.nih.gov/health_Information/Dietary_Reference_Intakes.aspx). These values are 2–3 times lower than the current average daily intake of sodium. On the other hand, high potassium intake has the opposite effect on urine calcium, namely it decreases it [16]. Whether this is due to potassium itself or to the fact that, in most cases, potassium in nature comes in the form of alkaline salts, such as potassium citrate, is not completely clear. In a small of group of children we were able to show that supplementation with potassium chloride reduced urine calcium [16]. However, it seems that in its alkali form, potassium has a more potent effect in decreasing urine calcium excretion [17]. The optimal daily potassium intake, provided mostly in the form of fruit, vegetable and dairy products, is 3.8 g at ages 4–8 years and 4.5 g at ages 9–18 years (http://dietary-supplements.info.nih.gov/health_Information/Dietary_Reference_Intakes.aspx). These values are 2–3 times higher than the current average intake. Interestingly, the opposite effects of sodium and potassium on urine calcium excretion are similar to their effects on blood pressure, namely excess sodium (especially in the form of NaCl) increases it, whereas high potassium intake lowers blood pressure [18]. A simple way to assess compliance with the above dietary recommendations related to sodium and potassium is by measuring the urine sodium/potassium ratio, which optimally should be below 2.5 [14].

In hypercalciuric stone formers, another important aspect of decreasing urine calcium is by changing the diet to be less acidic, by reducing animal-protein intake [19–21]. The metabolism of proteins results in formation of non-volatile acids which are buffered by bicarbonate released from bone, through bone resorption, which, when excessive, results in osteopenia and hypercalciuria [20, 21]. Furthermore, the acidic environment results in decreased urine citrate [21]. Indeed, as shown by Jehle et al. [22], treatment of adults on an acidogenic Western diet with potassium citrate increased bone mass, decreased calciuria, and increased citraturia. The natural source of potassium citrate is fruit and vegetables [13].

Whereas, in the past, dietary calcium restriction or binding with cellulose-phosphate was used to decrease absorptive hypercalciuria, this maneuver is not practiced any more, due to the fact that it results in increased oxalate absorption in the gut and has the potential to affect the bone adversely [19, 23]. Other nutrients, such as sucrose, fructose and high-dose vitamin C, may be associated with higher risk for kidney stone disease, whereas phytate and magnesium may decrease it [24]. As with other nutrients, it seems reasonable to assume that, especially in children, one should aim at RDA intake; however, these components of nutrition still require more research [25].

The issue of dietary oxalate restriction in patients with calcium oxalate stone is still debatable. Whereas some support this approach [26], others found no association between oxalate (and spinach) with higher risk for stones [27]. Perhaps a more clear differentiation should be made between patients with hypercalciuria and those with absorptive hyperoxaluria as the etiology for formation of calcium oxalate stones, with potentially more value to dietary oxalate restriction in the latter group (vide infra). On the other hand, calcium supplementation, when provided with meals, actually lowers oxalate absorption from the gut and its amount in the urine. This may be associated, though, with an increase in urine calcium [28].

It is strongly recommended that a nutritionist be involved to provide the family with the specifics on how to achieve the above nutritional goals. One should keep in mind that nutritional habits cannot be changed overnight, and a gradual change may be more tolerable and successful. The role of nutrition in other disease entities will be discussed later.

## Pharmacological intervention

### Calcium stones

#### Hypercalciuria and hypocitraturia

Treatment of patients with calcium stones due to idiopathic hypercalciuria with potassium (K)-citrate has the dual advantage of decreasing urine calcium and increasing urine citrate. Furthermore, it improves the bone mineral status of these patients [22, 29]. Naturally, K-citrate is the drug of choice in patients with hypocitraturia. It is important though to prevent the urine pH from becoming too alkaline thus promoting formation of calcium phosphate stones [30]. As always, a pH meter rather than a dipstick should be used when one is assessing urine pH.

Thiazides are time-proven preparations for the treatment of hypercalciuria. Interestingly, though, thiazides decrease urine citrate excretion, probably by the induced hypokalemia [29]. It thus may be advantageous to use a combination of thiazides plus K-sparing diuretics such as amiloride. The effect of the latter on urine citrate has not yet been proven; however, the combination thiazide/amiloride may have another advantage of further lowering urine calcium excretion compared with thiazides alone [31]. Another option to augment the hypocalciuric effect of thiazides, avoid hypokalemia and increase urine citrate is to use thiazides and K-citrate in combination [17].

In some patients with calcium oxalate stones, the results of chemical analysis of 24 h urine are normal, as is the anatomy of the urinary system. There could be several reasons why these patients develop stones, among them (a) abnormalities in less common inhibitors such as magnesium (Mg), stone-inhibiting urine proteins, or other yet unknown inhibitors; (b) it might be that, although each individual chemical is within its reference range, closer inspection may show that calcium and oxalate are at their upper limit of normal, while citrate is at the lower limit of normal; thus, their product supports stone formation; (c) one need to keep in mind that stone formation depends more on the concentration of the chemicals than on their quantity; hence, low urine output will increase the activity product of CaOx, even if the quantity of each individual chemical is within the normal range. The initial treatment of these patients should be non-pharmacological, and medications such as potassium citrate and thiazides should be added if the patient continues to form stones. This situation illustrates the relative greater importance of stone analysis than that of urine chemistry.

More commonly seen in adults rather than in children are calcium stones resulting from tubular “leak” of phosphate [32]. The latter lead to mild hypophosphatemia, causing increased production of 1,25(OH)_2_-vitamin D and, consequently, increased calcium absorption from the gut. The diagnosis is made by establishing the presence of low tubular threshold for phosphate, and the treatment is with phosphate preparations.

The growing interest in recent years in the bone–stone connection has revealed that approximately one-third of hypercalciuric children have decreased bone mineral density [33]. Recently, Heller et al. [34] showed that, in adults with absorptive hypercalciuria, bone mineral density was low and histomorphology showed increased bone resorption. Treatment of these patients with alendronate, an oral bisphosphonate preparation, decreased urine N-telopeptides, a marker of bone resorption, and calciuria. Not less importantly, patients reverted from a negative calcium balance to a positive one. A recent preliminary report showed similar beneficial effects of alendronate on bone and urine calcium excretion in children with osteopenia and calcium stone disease [35]. Further studies are needed with regard to the potential use of bisphosphonates in cases of hypercalciuria due to excessive bone resorption.

#### Hyperoxaluria

1. Primary hyperoxaluria
  The traditional treatment of primary oxaluria includes high fluid intake, thiazide diuretics, magnesium oxide, citrate and pyrophosphates. However, while all decrease the activity product of CaOx, none addresses the issue of hyperoxaluria per se. Owing to the fact that the major load of oxalate in these patients is due to the hepatic enzymatic defect, there is little rationale to recommend extreme restriction of dietary oxalate. However, it may make sense for the patients and their families to exercise avoidance of high oxalate foods (www.ohf.org/treatment.html).
  It has been known for some time that 30–50% of patients with type I disease lower their urine oxalate in response to treatment with pyridoxine (vitamin B_6_). Only in recent years has it become evident that it is the patient’s genotype that dictates such response, seen only in those with the commonest mutant allele, G630A [36]. Pyridoxine is effective in individuals both homozygous and heterozygous for these alleles, though the specific mechanism of the vitamin’s action under these circumstances is yet unknown. A novel approach to the treatment of hyperoxaluria might be achieved with oral intestinal colonization with *Oxalobacter formigenes*, which not only degrades intestinal oxalate but also enhances colonic secretion of endogenously produced oxalate, resulting in decreased blood and urine oxalate levels [36, 37]. In patients who develop chronic renal failure and end-stage kidney disease, aggressive dialysis, followed by liver and kidney transplantation, is currently the best available approach.
2. Absorptive hyperoxaluria
  Absorptive hyperoxaluria can be idiopathic (namely due to yet unknown causes) or secondary to abnormalities in the gastrointestinal (GI) system, mostly associated with fat malabsorption. The treatment of this condition involves correction of the basic GI tract anomaly, restricted dietary oxalate intake, and, at times, increased calcium intake. Assistance in consulting the patient on oxalate restriction can be found at The Oxalosis and Hyperoxaluria Foundation website (www.ohf.org/docs/Oxalate2004.pdf). Supplemental calcium is given to compensate for calcium captured by the unabsorbed fat and therefore not available to bind oxalate.
  As discussed above, oral ingestion of *Oxalobacter formigenes* may have the same effect as in primary hyperoxaluria. The ingestion of lactic acid bacteria, which degrade oxalate in vitro, resulted in mixed results. While found to be ineffective in patients with idiopathic absorptive hypercalciuria [38], others found it to be effective in decreasing urine oxalate in patients with secondary absorptive hyperoxaluria due to chronic fat malabsorption [39].

### Cystinuria

Healthy individuals excrete fewer than 30 mg (0.13 mmol) of cystine per day, whereas homozygote patients excrete between 400 mg and 3,000 mg (1.7 and 13 mmol) day. The goal of treatment is to keep cystine soluble at a concentration below 250 mg (1 mmol)/l. This means that a patient who excretes 750 mg (3 mmol) cystine per day needs to have a urine volume of 3 l in order to maintain the urinary cystine soluble. The fluid intake should be distributed throughout the day and night, which means that the patient should have significant fluid intake before retiring to bed and, ideally, also at least one additional intake during sleeping hours. In reality, some patients may not be able to achieve such a demanding goal. As cystine solubility increases dramatically in alkaline urine, urine pH should be kept between 7.0 and 7.5. Besides beverages and food containing alkali, the optimal agents to alkalinize the urine are potassium citrate and potassium bicarbonate. One may also consider the use of acetazolamide; however, the use of carbonic anhydrase inhibitors always carries the risk of too alkaline urine associated with hypocitraturia, which may result in formation of calcium phosphate stones. Urinary excretion of cystine correlates with dietary sodium intake; thus, a diet low in sodium is recommended, as well as avoidance of sodium-based alkaline preparations.

In some patients the aforementioned intervention may suffice to prevent the formation of new stones, but, in many others, additional, specific, therapy is required [13]. Both, D-penicillamine and tiopronin are sulfhydryl compounds which cleave cystine into two cysteine-disulfide moieties that are 50-times more soluble than cystine. Although the treatment with D-penicillamine is effective, it carries a high incidence of serious side effects. If needed to be used long-term, it should be supplemented with pyridoxine (vitamin B_6_), 25–50 mg/day, because of the anti-pyridoxine effect of the medication. The tendency nowadays is to use first tiopronin, which seems to have a lower incidence of side effects. Captopril, which is a sulfhydryl agent, has been used with mixed results in cystinuria. The recommended dose in adults is 75–150 mg per day. Because of its potential hypotensive effect, some recommend trying it after all other means have been unsuccessful.

### Infection-related urolithiasis

Such stones have become less common but are still seen at times, especially in cases of underlying anatomic predispositions. Infection stones are mostly composed of magnesium ammonium phosphate (MgNH_4_PO_4_-6H_2_O), also known as struvite, and carbonate apatite (Ca_10_[PO_4_]_6_CO_3_). Struvite stones can develop very rapidly and, at times, form a cast in the pelvocaliceal system, known as “staghorn calculus”. The stones are formed in the presence of bacteria such as *Proteus* spp., *Staphylococcus aureus*, *Klebsiella* spp. and others which produce urease, causing the breakdown of urea to ammonium and bicarbonate. The latter results in alkaline urine which promotes the formation of these stones. One of the essential management strategies in infection-related stone is to sterilize the urinary tract. Often, this is possible only after the infected stone has been removed. Prevention of recurrence of such stones requires correction of the underlying anatomic abnormality and protection from infection. In addition, urine acidification with acid-phosphate preparation may keep the milieu in the kidney unfavorable for formation of such stones. Finally, acetohydroxamic acid, a urease inhibitor, has been successfully used in adults, but, due to its potential serious side effects, has not been used in children [40].

### Uric acid stones

The formation of uric acid stones is due to either high rates of urinary urate excretion or persistently low urine pH, or a combination of the two. The first line of treatment is urine alkalinization, optimally by potassium citrate. In case there is a need to lower urate excretion, dietary purine restriction is indicated (www.dietaryfiberfood.com/purine-food.php), and, if needed, allopurinol can be added. If, in spite of good biochemical control and optimal urine pH, the patient continues to generate stones, urine xanthine level should be checked for a possible etiology, as the level may rise significantly, secondary to the treatment with allopurinol [14]. One should keep in mind the existence of stones made of both uric acid and calcium. In such cases, potassium citrate would be the first line of treatment, while thiazides and allopurinol can be used if indicated.

### 2,8-Dihydroxyadeninuria

This autosomal recessive disorder is caused by a deficiency in adenine phosphoribosyl transferase, leading to excessive formation of 2,8-dihydroxyadenine. The stones are very similar to uric acid stones, and specialized analysis of chemical stones is required to identify them. A strong clue to this diagnosis are normal serum and urine concentrations of uric acid (however, such conditions can also occur in uric acid lithiasis caused by chronically acidic urine). These stones are amenable to preventive treatment with allopurinol and a low purine diet; however, in contrast to uric acid stones, their solubility decreases in the face of alkaline urine [13].

## Other etiologies of stone disease in childhood

In some patients the development of stones is secondary to the presence of another condition or disease causing urinary biochemical abnormalities which promote the development of stones [41]. For instance, immobilization, hyperparathyroidism, and sarcoidosis frequently cause hypercalciuria; distal renal tubular acidosis (dRTA) hypercalciuria and hypocitraturia; and myeloproliferative disorder hyperuricosuria. Additionally, stones can be caused iatrogenically. Loop diuretics, corticosteroids, and excess vitamin D cause hypercalciuria; ketogenic diet hypocitraturia and hyperuricosuria; and parenteral nutrition in premature infants hyperoxaluria. In other instances it is the medication itself, or its metabolites, which precipitate and form stones, as in the case of indinavir, ceftriaxone, felbamate and others. A complete list of the conditions, diseases, and medications associated with stone formation is beyond the scope of this review, and more details can be found elsewhere [13, 41]. The common denominator of all these etiologies is that identification and successful treatment of the basic disorder (i.e. hyperparathyroidism, dRTA) or discontinuation of the offending medication will protect the patient from developing new stones.

## Questions

### (Answers appear after the reference list)

1. The minimum urine flow (in milliliters per kilogram per hour) which protects from calcium oxalate supersaturation is
  - >0.5
  - >0.75
  - >1.0
  - >1.5
2. The recommended maximum daily sodium intake between the ages 9 years and 18 years is (in milligrams per day)
  - 2,000
  - 2,300
  - 2,800
  - 3,000
3. The recommended daily intake of potassium between the ages 9 years and 18 years is (in milligrams per day)
  - 2,900
  - 3,500
  - 3,900
  - 4,500
4. In treating cystinuria the goal is bringing urine cystine concentration below (in milligrams per liter)
  - 250
  - 350
  - 500
  - 750
5. Dietary NaCl
  - decreases the patient’s acidic milieu
  - increases urine citrate
  - increases the patient’s acidic milieu
  - has no effect on acid–base status
